# Management of respiratory tract exacerbations in people with cystic fibrosis: Focus on imaging

**DOI:** 10.3389/fped.2022.1084313

**Published:** 2023-02-06

**Authors:** Nicholas Landini, Pierluigi Ciet, Hettie M. Janssens, Silvia Bertolo, Mirco Ros, Monica Mattone, Carlo Catalano, Fabio Majo, Stefano Costa, Andrea Gramegna, Francesca Lucca, Giuseppe Fabio Parisi, Luca Saba, Harm A. W. M. Tiddens, Giovanni Morana

**Affiliations:** ^1^ Department of Radiological, Oncological and Pathological Sciences, Policlinico Umberto I Hospital, “Sapienza” Rome University, Rome, Italy; ^2^ Department of Radiology and Nuclear Medicine, Erasmus MC – Sophia, Rotterdam, Netherlands; ^3^ Department of Radiology, University Cagliari, Cagliari, Italy; ^4^ Department of Pediatrics, division of Respiratory Medicine and Allergology, Erasmus MC – Sophia Children’s Hospital, University Medical Center Rotterdam, Rotterdam, Netherlands; ^5^ Department of Radiology, S. Maria Ca’Foncello Regional Hospital, Treviso, Italy; ^6^ Department of Pediatrics, Ca’Foncello S. Maria Hospital, Treviso, Italy; ^7^ Pediatric Pulmonology & Cystic Fibrosis Unit Bambino Gesú Children's Hospital, IRCCS Rome, Rome, Italy; ^8^ Department of Pediatrics, Gaetano Martino Hospital, Messina, Italy; ^9^ Department of Pathophisiology and Transplantation, University of Milan, Milan, Italy; ^10^ Respiratory Disease and Adult Cystic Fibrosis Centre, Internal Medicine Department, IRCCS Ca’ Granda, Milan, Italy; ^11^ Regional Reference Cystic Fibrosis Center, University Hospital of Verona, Verona, Italy; ^12^ Pediatric Pulmonology Unit, Department of Clinical and Experimental Medicine, University of Catania, Catania, Italy

**Keywords:** cystic fibrosis, respiratory tract exacerbations, inflammation, imaging, lung function, computed tomography, magnetic resonance imaging, chest radiography

## Abstract

Respiratory tract exacerbations play a crucial role in progressive lung damage of people with cystic fibrosis, representing a major determinant in the loss of functional lung tissue, quality of life and patient survival. Detection and monitoring of respiratory tract exacerbations are challenging for clinicians, since under- and over-treatment convey several risks for the patient. Although various diagnostic and monitoring tools are available, their implementation is hampered by the current definition of respiratory tract exacerbation, which lacks objective “cut-offs” for clinical and lung function parameters. In particular, the latter shows a large variability, making the current 10% change in spirometry outcomes an unreliable threshold to detect exacerbation. Moreover, spirometry cannot be reliably performed in preschool children and new emerging tools, such as the forced oscillation technique, are still complementary and need more validation. Therefore, lung imaging is a key in providing respiratory tract exacerbation-related structural and functional information. However, imaging encompasses several diagnostic options, each with different advantages and limitations; for instance, conventional chest radiography, the most used radiological technique, may lack sensitivity and specificity in respiratory tract exacerbations diagnosis. Other methods, including computed tomography, positron emission tomography and magnetic resonance imaging, are limited by either radiation safety issues or the need for anesthesia in uncooperative patients. Finally, lung ultrasound has been proposed as a safe bedside option but it is highly operator-dependent and there is no strong evidence of its possible use during respiratory tract exacerbation. This review summarizes the clinical challenges of respiratory tract exacerbations in patients with cystic fibrosis with a special focus on imaging. Firstly, the definition of respiratory tract exacerbation is examined, while diagnostic and monitoring tools are briefly described to set the scene. This is followed by advantages and disadvantages of each imaging technique, concluding with a diagnostic imaging algorithm for disease monitoring during respiratory tract exacerbation in the cystic fibrosis patient.

## Introduction

Cystic fibrosis (CF) is a systemic disease in which lung involvement is the major cause of morbidity and mortality (1). Although improvements in patients' management and therapeutic strategies have increased the life expectancy of people with CF (PCF), it remains lower than in the general population (1). Respiratory tract exacerbations (RTE) contribute significantly to lung morbidity and disease progression in PCF; recurrent RTE during childhood are associated with lung function deterioration, analysed, for example, *via* spirometry, and structural abnormalities, such as bronchiectasis, detected by computed tomography (CT) (2). The frequency of RTE increases with patient age. Moreover, older PCF require longer antibiotic treatment, which impacts adversely on both patient quality of life and costs for the healthcare system (3). Moreover, most PCF who experienced RTE do not recover their pre-exacerbation lung function (3).

The etiology of RTE is not completely understood. Infections by new pathogens (e.g., viruses) and overgrowth of preexisting airway flora (mostly bacteria) can determine the nature of acute infection and, since pathogen determination is not always possible *via* sputum sample, treatment may be complex. While invasive approaches, such as bronchoalveolar lavage (BAL), provide higher sensitivity and specificity (4), they are not routinely performed in all CF centers.

The treatment of RTE is not standardized either (2) and there is no international consensus on its optimal duration. Current RTE treatment, especially antibiotic therapy, lasts on average 14 days, with conflicting evidence for the use of prolonged treatment intervention (2). Thus, clinicians have no objective tools or guidelines to support clinical decision making, relying, instead, on improvement of pulmonary function tests (PFTs), e.g. forced expiratory volume in the first second (FEV_1_), and relief of symptoms (i.e., cough and sputum production) (5).

For all these reasons, early diagnosis of RTE is crucial for timely initiation of therapy, as well as optimizing treatment efficacy. As recently highlighted in national guidelines (1), imaging techniques can improve both RTE detection and monitoring of therapy, providing information that may support clinicians with patient care.

Although evidence for its use remains controversial, chest radiography (CR) is the most frequently used imaging technique for RTE detection (6). The gold standard for the assessment of structural lung abnormalities in PCF, however, is computed tomography (CT), especially after the introduction of the low and ultra-low dose CT protocols (7). Despite its advantages, the use of CT remains limited for short-term follow-up serial imaging—as used in RTE—largely due to concern regarding excessive radiation exposure.

Conversely, magnetic resonance imaging (MRI) offers a useful and safe alternative, especially for both inflammation and functional assessment (1), while lung ultrasound (LUS) has been recently proposed as a point-of-care diagnostic tool for repeatable bedside RTE monitoring (8).

This state-of-the-art review summarizes the clinical challenges of RTE in PCF with special focus on imaging. A short description of the definition of RTE, available diagnostic and monitoring tools, and current treatment options are firstly given to illustrate the clinical context. The review then discusses the advantages and disadvantages of respective imaging techniques, which incorporates a diagnostic imaging algorithm for monitoring PCF during RTE.

## Respiratory tract exacerbations definition

The Cystic Fibrosis Foundation defines RTE as “acute worsening of respiratory symptoms” requiring medical treatment, mainly represented by antimicrobial therapy and airway clearance through physiotherapy, often under hospitalization (9). The occurrence of RTE is a risk factor for decreased life quality (10, 11), increased medical resource utilization (12), and mortality (13, 14). Therefore, reduction in RTE rate and/or risk has become a robust efficacy measure for trials of chronic CF therapies (15).

Nonetheless, a shared and objective definition of RTE, exploitable as an outcome in clinical trials, is still lacking.

The easiest way to define RTE is to consider the beginning and end dates of antimicrobial treatment on the patient medical record, assuming that the treatment correlated with the onset and resolution of RTE. The choice of drug administration route (intravenous vs. oral) and intervention type (hospital at home vs. in-hospital stay) depend on the RTE severity (16, 17).

The need for more objective criteria for identifying RTE has been driven by the need to avoid variability in clinician assessment.

The dornase alfa Phase 3 study (16) defined RTE as a respiratory event treated with intravenous (IV) antimicrobials, where at least 4 out of 12 specific signs and symptoms were present. Following this publication, these criteria are referred to as the “Fuchs criteria” and are frequently used in both studies and clinical practice (Table 1). Other groups have proposed similar criteria to define RTE, such as the Rosenfeld criteria (18). Since the probability of requiring IV antimicrobial treatment for exacerbation is age- and lung-function-dependent (19) [unlike the overall rate of RTE subjectively assessed by clinicians (20)], the RTE definition has been expanded to include any antimicrobial treatment. This kind of definition has been recently used to explore the rate/risk of exacerbation in clinical trials on CF transmembrane conductance regulator (CFTR) modulators (21).

**Table 1:** Original fuchs criteria: at least 4 out of 12 of the following signs and symptoms must be present for respiratory tract exacerbation diagnosis.

Fuchs criteria
Increased cough
Change in sputum
New or increased hemoptysis
Increased dyspnea
Malaise, fatigue, or lethargy
Temperature above 38°C
Anorexia or weight loss
Sinus pain or tenderness
Change in sinus discharge
Change in physical examination of the chest
Decrease in pulmonary function by 10% or more from a previously recorded value
Radiographic changes indicative of pulmonary infection

## Clinical scenario and diagnosis

A challenging aspect of using the Fuchs criteria in clinical practice is that prescribed antibiotic therapy forms part of the diagnostic criteria for RTE as well as its treatment.

The selection of antibiotic therapy is governed by strong subjective influence, which greatly limits the applicability of the Fuchs criteria in daily practice. For example, in a clinical trial using hypertonic saline, Elkins et al*.* (22) compared a version of the Fuchs criteria based on symptoms (regardless of the prescribed treatment) with classic criteria, confirming the strong variability introduced by clinician opinion. This is unsurprising, since some of the original criteria, such as physical examination, may be nonspecific or present with various clinical scenarios, that can lead to different therapeutic approaches.

Therefore, an international group of experts (23) has suggested a modified version of the Fuchs criteria that involves the presence of two out of six signs or symptoms (Table 2). The following original criteria were removed: new or increased hemoptysis, temperature over 38°C, sinus pain or tenderness, change in sinus discharge and change in chest findings on physical examination; the latter was excluded because it was too difficult to define and standardize. However, although physical examination has low sensitivity and is difficult to standardize, it remains the first and most used method to diagnose RTE.

**Table 2:** Modified Fuchs criteria according to the European Consensus Group: at least 2 out of 6 of the following signs and symptoms must be present for respiratory tract exacerbation diagnosis.

Modified Fuchs criteria
Change in sputum volume or color
Increased cough
Increased malaise, fatigue, or lethargy
Anorexia or weight loss
Decrease in pulmonary function by 10% or more/radiographic changes
Increased dyspnea

Many gaps in our knowledge about the clinical management of the acute phase of RTE remain (3). Firstly, standardized diagnostic criteria might help define RTE in both daily practice and clinical research; possible nonbacterial and noninfectious etiologies, uncontrolled comorbidities and poor treatment adherence should also be carefully considered. Secondly, predictive biomarkers must be identified and implemented to confirm the RTE diagnosis and monitor treatment response. Thirdly, the establishment of agreement on standardized antibacterial therapy and its duration for RTE are areas of new research, which offer many implications for clinical practice (24). Then, although RTE increase in frequency and severity based on the degree of lung damage, there is still no means of structured assessment that can be used in the decision-making process for treatment selection. Finally, the therapeutic landscape of CF lung disease is being significantly altered following the arrival of new classes of CFTR modulators (25), where their long-term impact on CF progression and RTE frequency is still unknown. Whether RTE will play a different role in CF progression in the near future is a key topic to be addressed by further research.

## Physical examination

During RTE, findings of the physical examination are part of the objective measures that most scoring tools use combined with (subjective) symptoms. However, these findings are frequently subject to low interobserver repeatability, e.g., changes on auscultation (26). The odds of a clinician-diagnosed RTE have recently been shown to increase when associated with reported symptoms and a drop in forced expiratory volume (FEV_1_). However, evaluating FEV_1_ decline and subsequent RTE management in non-cooperative children can be challenging (27).

Physical examination of the chest is a Fuchs criterion for RTE diagnosis (16) and part of the scoring system in the multivariate model developed by Rosenfeld; however, in the study by Rosenfeld et al., the most important features in determining the presence of RTE were symptoms and clinical history rather than physical examination and laboratory results (18).

Most scoring tools are barely used in a clinical setting, while lung examination findings remain central to the clinical practice of any clinician. However, the last two years of the COVID-19 pandemic have reduced reliance on traditional diagnostic criteria, especially the physical examination. The impossibility of visiting patients made telehealth more prevalent, and RTE had to be defined without examination or spirometry (28).

In the absence of any physical examination, symptoms recalled by PCF or caregivers are a direct and fundamental source of information. Patient-reported outcomes represent the first attempt at standardizing subjective symptoms and their incorporation as additional endpoints in scoring tools and clinical practice is becoming a developing area in RTE definition and monitoring (29).

## Pulmonary function tests and functional biomarkers

Spirometry has always been considered the main pulmonary function test (PFT) in PCF. In particular, the forced expiratory volume in the first second (FEV_1_) is the most used parameter to assess the severity of lung disease, measure outcomes in clinical trials, evaluate therapeutic response and refer patients for lung transplantation (16, 30, 32). The measure can also be combined with clinical criteria to identify RTE and assess its trend over time or evaluate therapy duration and efficacy (16, 32, 33). Most authors recognize an FEV_1_ decline >10% as an indicator of RTE, and it is used as a criterion for initiating oral or IV antibiotic therapy (16, 34). This explains the need for spirometry as a cornerstone of the annual check-up performed at CF centers (35, 36).

On the other hand, PFTs parameters assessed by spirometry are also used to determine the duration of antibiotic therapy during RTE, but data from the literature are controversial.

Redding et al. monitored the lung function of 17 children with CF every day during a 14-day hospitalization to undergo IV antibiotic therapy, but failed to demonstrate an optimal duration of treatment because the FEV_1_ response was highly variable (36); similar results were observed in the retrospective study by Rosenberg and Schramm (37).

Conversely, a retrospective study by Collaco et al. suggested that the duration of antibiotic therapy during RTE can be reduced to 7–10 days when a stable improvement in FEV_1_ is evident within this period (38). More recently, Stephen et al. assessed daily FEV_1_ in an adult CF population with RTE, demonstrating that achieving an FEV_1_ increase of >10% takes an average of 6 days; however, the result variability was so large that the authors could not assess the optimal duration of antibiotic therapy. Therefore, other independent variables of PFT have been suggested to determine the therapy duration (40).

Moreover, RTE may result in an irreversible loss of lung function, with an FEV_1_ that does not always return to pre-exacerbation values (39, 40), although the reason that lies behind this eventuality remains unclear.

Besides, PFTs have some further intrinsic limitations, since there may be a big discrepancy between structure and function (41), e.g., preserved lung function may disguise extensive bronchiectasis. Furthermore, young children (below 6 years) cannot perform the forced maneuver for traditional PFTs. These observations emphasize the need for new tools to ensure a more sensitive assessment of RTE, beyond clinical and spirometric analyses (42).

New PFTs correlated with structural changes have been recently investigated, including the multiple breath washout test, which allows the assessment of the lung clearance index (LCI). The LCI is a parameter of ventilatory inhomogeneity which is currently recognized as the most sensitive index for identifying small airway obstruction (43–45). Despite intra-individual variability of up to 15%, higher values of LCI may be a telltale sign of RTE (46, 47). Walicka et al. proposed a lower cut-off to define RTE based on LCI, where the LCI threshold should be reduced to 10%, as this would increase its sensitivity compared with conventional spirometry and facilitate calculations in clinical practice (48). They also showed that RTE are associated with a 9% worsening of LCI even at the end of treatment (48), a percentage that is reduced to 3% according to Perrem et al. (46). Furthermore, more recent studies indicate the LCI to be a far more sensitive parameter than FEV_1_, with rapid LCI changes being seen at the onset of RTE. The combined assessment *via* spirometry and LCI is superior to diagnose and monitor PCF during RTE (47–49). However, LCI is not currently used as a standard of care because it requires specialized personnel and equipment and is rather time-consuming.

## Other biomarkers

### Infection

Respiratory tract exacerbations in CF are treated with antibiotics directed against microorganisms found in the airways. Because infections by microorganisms, such as *Pseudomonas aeruginosa,* accelerate lung function decline in PCF, eradication therapy must be quickly initiated.

Microbiological cultures from airways are therefore taken regularly and represent an important biomarker for the management of CF lung disease; samples can be collected from BAL, sputum or the upper airways, with decreasing reliability of accuracy for finding microorganisms in the lower airways (50, 51). Bronchoalveolar lavage is considered the gold standard for assessing the presence of microorganisms in the lungs. However, the nature of the procedure—which is invasive, requiring bronchoscopy under anesthesia—means that it is not routinely done in all CF centers. Sputum sampling is a good alternative, but young children cannot always cough up enough sputum for cultures. Furthermore, cultures obtained from the upper airways often do not reflect the microorganisms residing in the lungs.

As a result of these limitations, research has been focused on finding other ways to detect microorganisms in the lungs. For example, the analysis of volatile organic compounds in exhaled breath is a promising tool; this technique can discriminate between positive and negative *Pseudomonas* cultures in PCF (52). However, most research on other biomarkers is still in the developmental phase.

### Inflammation

There has been an increase in our understanding of the role of inflammation in the pathophysiology of CF lung disease. Although it is known that a vicious circle of infection and neutrophilic inflammation causes structural damage to the lungs, a debate exists regarding which comes first. By 1995 it was shown that, despite the absence of microorganisms in BAL cultures, there were signs of inflammation in the lungs in patients as young as 4 weeks. It has also been demonstrated that increased neutrophil elastase (NE) and increased neutrophils in BAL infants are risk factors for developing bronchiectasis at a later age (53, 54). Research on RTE biomarkers—for example, as found in sputum, serum and exhaled breath condensate (EBC)—in CF has therefore focused on assessing inflammation to predict exacerbations (55).

Numerous studies have investigated sputum as a source of biomarkers in CF. Existing sputum biomarkers with higher scientific reliability include interleukin (IL)-8 and NE, since their levels increase during RTE and decrease after treatment (56–58). A recent extensive review by the biomarker Special Interest Working Group of the European Cystic Fibrosis Society–Clinical Trials Network Standardization Committee stated that sputum biomarkers hold promise as a direct measure of inflammation, with NE, IL-8, tumor necrosis factor alpha, and IL-1β showing that validity and responsiveness should be considered as outcome measures in clinical trials. However, variability in study design and sampling methods has limited their use in clinical practice. Future focus should therefore be on creating standards for the collection, storage, and analysis of sputum biomarkers (59).

EBC analysis represents an innovation that has great potential for understanding the biochemical and metabolic mechanisms of respiratory disease. The EBC is the fluid obtained in a condenser by cooling exhaled air during current volume breathing. In PCF, an important contribution to pulmonary damage originates from an increasing imbalance between oxidant and antioxidant molecules in the airways. For example, extracellular glutathione transport may be altered in PCF, contributing to the loss of an important airway antioxidant defense mechanism. Furthermore, high levels of malondialdehyde and 8-isoprostane, two biomarkers of oxidative stress, have been found during RTE in the EBC of PCF (60, 61). However, in another study, 8-isoprostane and nitrite in EBC failed to show sufficient sensitivity for RTE prediction (62). A further study has reported increased NE in the EBC of children with CF. However, nitric oxide, IL-17, IL-8, e-cadherin, NE, or leukotriene B4 levels in the EBC of CF children were not related to *P. Aeruginosa* infection, FEV_1_ levels, or hospital admission in the last year (63). So far, sampling difficulties and variable results have failed to prove the usefulness of EBC in clinical practice.

**Figure 1:**
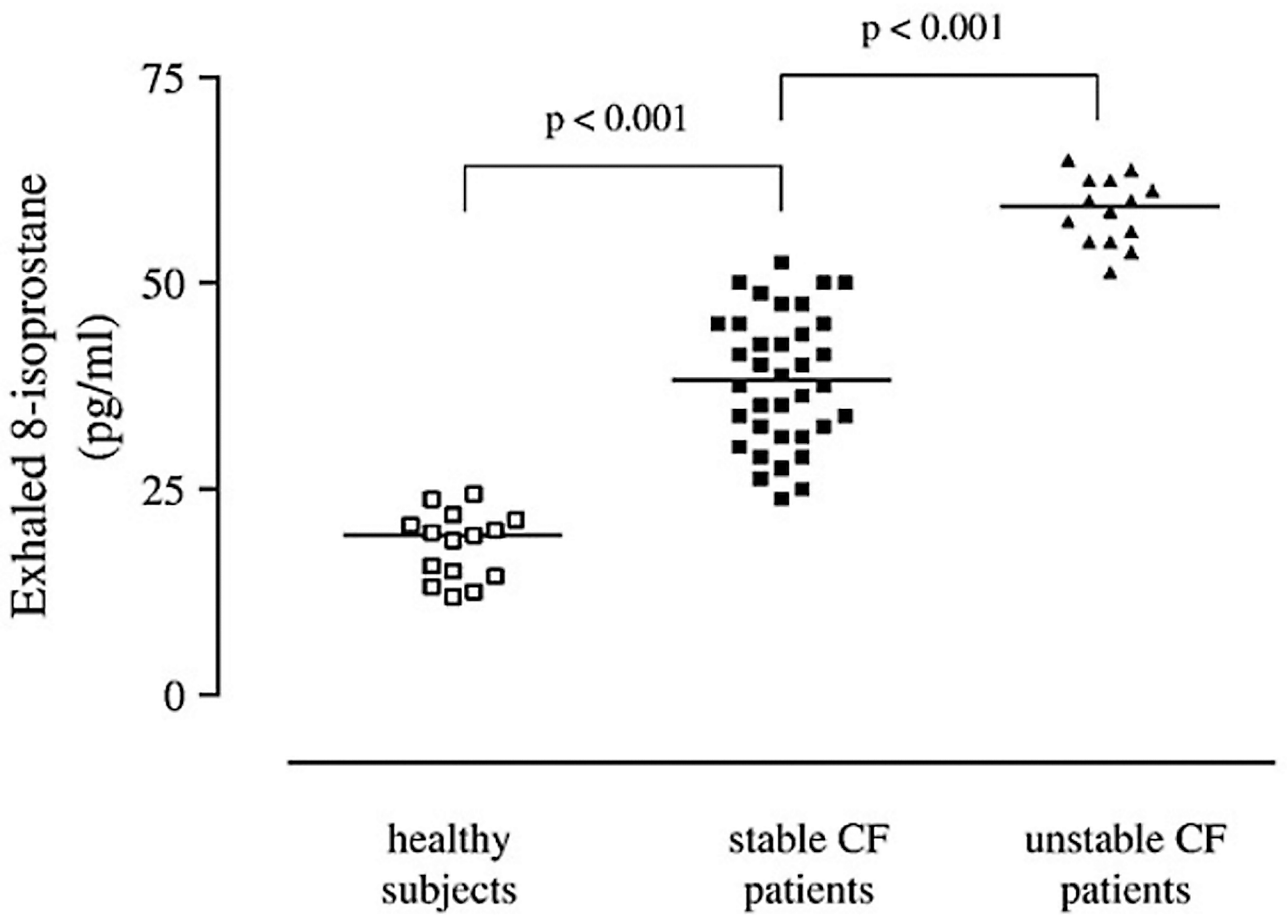
Adapted from “Exhaled 8-isoprostane and prostaglandin E(2) in patients with stable and unstable cystic fibrosis”: 8-Isoprostane concentrations in exhaled breath condensate in healthy subjects (open squares) and patients with stable (filled squares) and unstable (filled triangles)cystic fibrosis. Horizontal bars represent median values (61).

Finally, metabolomic studies provide potentially interesting data. In this regard, a recent systematic review identified two potential metabolites (4-hydroxycyclohexylcarboxylic acid and lactic acid) that could predict RTE when measured in EBC and four other metabolites (sputum lactic acid, nitrate, plasma arginine, and methionine) with a high sensitivity for RTE diagnosis (Figure 1) (64).

Regarding blood biomarkers, a recent meta-analysis concluded that there is reasonable evidence to support their application in RTE diagnosis but further studies are needed to demonstrate the usefulness of this approach (65). Although the most used serum marker in CF is C-reactive protein, there is insufficient evidence that levels correlate with RTE severity (66). More interestingly, levels of calprotectin – a neutrophil-derived protein – increase during RTE and decrease after treatment, and apparently correlate with RTE prediction more sensitively than C-reactive protein (67, 68). Other biomarkers under study include serum amyloid A, IL-1Ra, and some cleavage products of the endogenous respiratory tract proteins produced during episodes of infection, e.g., secretory leukocyte protease inhibitor, elafin, and LL-37 (69, 70).

In conclusion, the most promising predictive inflammatory biomarkers for RTE in PCF appear to be NE, IL-8, tumor necrosis factor alpha, and IL-1 β in sputum and calprotectin in serum. The metabolomic analysis of EBC, sputum, and serum has shown interesting results but needs further investigation.

## Imaging techniques

Imaging plays a key role in RTE management by its ability to provide structural and functional information. The advantages and disadvantages of the imaging techniques currently available are discussed in the following paragraphs and summarized, along with the other diagnostic methods, in Table 3.

**Table 3:** Advantages and disadvantages for each respiratory tract exacerbation evaluation method.

Method	Advantages	Disadvantages
Physical examination	Fast Accessible	Interobserver variability No standardized assessment No direct inflammation assessment No microorganism detection
FEV_1_	Functional evaluation Accessible Reference values	Poor sensitivity Lack of structural and inflammation assessment No microorganism detection Not feasible with uncooperative patients
LCI	Ventilation inhomogeneity assessment Higher sensitivity than FEV_1_	Lack of structural and inflammation assessment No microorganism detection Not feasible with uncooperative patients Limited availability Time-consuming
BAL	Microorganism detection^a^ Inflammatory markers quantification	Lack of structural and functional assessment Invasive procedure requiring anesthesia Operator dependence Limited availability
EBC	Inflammation assessment	Lack of structural and functional assessment No microorganism detection Limited availability
Sputum biomarkers	Microorganism detection Inflammation assessment Availability	Lack of structural and functional assessment Not feasible with uncooperative patients Low sensitivity because of sampling error
Airway swaps	Microorganism detection Inflammation assessment Availability	Poor accuracy because of the discrepancy between upper and lower airway flora Lack of structural and functional assessment
Blood biomarkers	Inflammation assessment Availability	Invasive Lack of structural and functional assessment No microorganism detection
CR	Accessible Low-cost Low radiation dose	Poor sensitivity No inflammation assessment No microorganism detection Limited functional evaluation
CT	Structural and functional assessment^a^ Availability	Higher radiation exposure than CR Indirect inflammation assessment No microorganism detection
MRI	Structural and functional assessment Inflammation assessment No radiation exposure	No microorganism detection Higher costs than CT Lower accessibility than CT Longer scan time than CT Sedation/general anesthesia needed in uncooperative patients
LUS	Low cost High accessibility No radiation exposure	Bidimensional technique limited to the lung surface Limited inflammation assessment No microorganism detection No functional evaluation assessment (except diaphragm function) High operator dependence

BAL, bronchoalveolar lavage; CR, chest radiography; CT, computed tomography; EBC, exhalate breath condensate; FEV_1_, forced expiratory volume in the first second; LCI, lung clearance index; LUS, lung ultrasound examination; MRI, magnetic resonance imaging.

^a^ Current standard.

### Chest radiography

CR is usually the first imaging tool utilized for detecting RTE due to its ready availability, low cost, and lowest radiation exposure compared with CT (1). A recent review has given dose ranges for CR and CT imaging in PCF (7). When comparing the effective dose of current low-dose CT protocols for PCF to a single projection CR (either anterior-posterior or posterior-anterior) the dose range of CT is in the order of 15–30 CRs (7). This range is expected to further decrease by the introduction of ultra-low-dose protocols (7).

**Figure 2:**
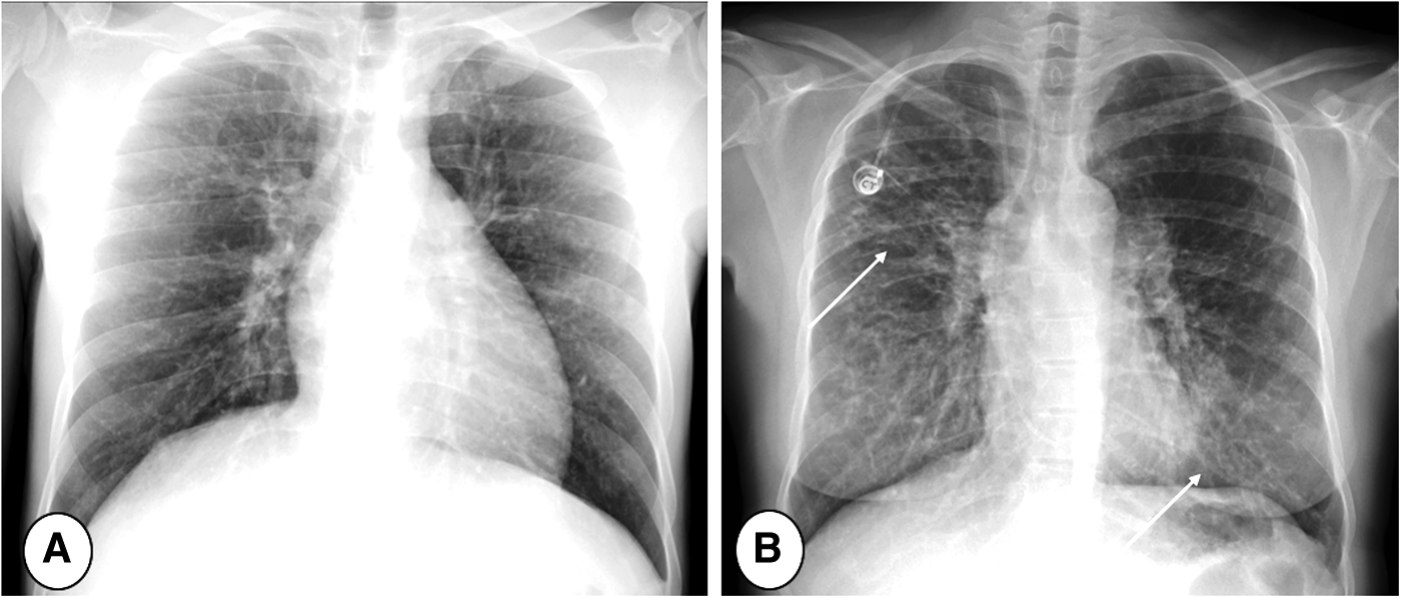
Posterior/anterior chest radiographs in (**A**) mild and (**B**) severe cystic fibrosis lung disease. Note the progressive increase of bronchial pathology in the subject with severe disease (B, arrows). The more severe the disease, the more difficult the detection of new abnormalities, especially during respiratory tract exacerbation.

**Figure 3:**
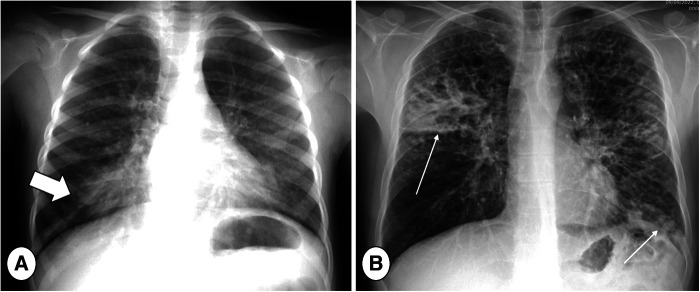
Posterior-Anterior (PA) chest radiograph in mild (**A**) and severe (**B**) cystic fibrosis lung disease during respiratory tract exacerbation. Note large consolidation in the right lung base in image A indicating respiratory tract exacerbation (thick arrow) and bilateral consolidations in the right upper lobe and left lung base (B, thin arrows).

However, CR has low sensitivity and specificity in both detecting and following up on new lung alterations. Since lung abnormalities may not differ between patients with and without RTE, nor before and after therapy (6), because of differing disease severities, new CR abnormalities can be very difficult to detect among severe parenchymal changes (70) (Figure 2). Moreover, some alterations are focal in nature and not directly visible in a single projection, being hidden in blind spots, e.g., lung apices and retrocardiac regions (71). Thus, the use of CR is mainly supported by the need to exclude major complications, such as pneumothorax (6), or for assessment of gross abnormalities (Figure 3).

More recently, dynamic CR has been proposed for RTE monitoring. FitzMaurice et al. demonstrated improving speed and range of the diaphragmatic movement in PCF during RTE following treatment (72). Dynamic CR data were acquired over 10 seconds; with machine learning software, diaphragm motion and lung density were extracted during tidal breathing acquisition and from full inspiration to passive end-expiration. The results of this single-center study, however, need further confirmations to assess the possible role of this technique in RTE evaluation.

### Computed tomography

Given its ability to provide a detailed view of regional airway and parenchymal changes, CT remains the tool of choice for assessing structural changes in CF lung disease alongside CR. The technique currently offers the best spatial resolution of all imaging modalities. Common CT scanners have a resolution in the order of one millimeter (mm), which allows detection of airways as small as 2 mm in diameter. For an adult without lung disease, this degree of resolution means that during an inspiratory CT scan airways up to the 13th generation can be detected. This is less for preschool children when only more central airways up to the 9th generation can be seen (73, 74). However, inspiratory and expiratory CT scans reveal data on ventilatory alterations and show higher sensitivity than spirometry in detecting early small airways disease (70). Therefore, well-established scores of lung involvement in PCF can be useful during RTE.

**Figure 4:**
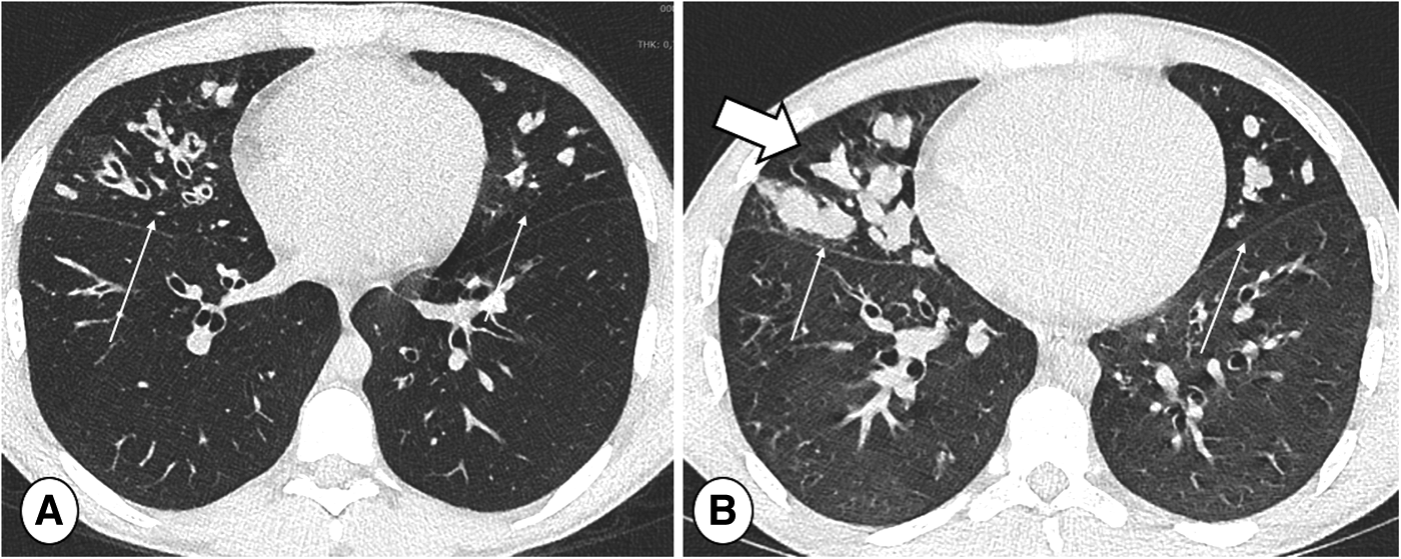
End-inspiratory CT of a cystic fibrosis patient (**A**) without and (**B**) with respiratory tract exacerbation, lung window. Note bronchiectasis and mucus plugs, especially in the middle lobe and lingula (thin arrows). The only difference indicating RTE is an increase in central mucus plugs (thick arrow).

Davis et al., using the modified Brody score, demonstrated that CT can reveal areas of active inflammation during RTE (which is sometimes used to guide sampling) which improves after treatment (75). Moreover, CT may be helpful in the stratification of risk for RTE. For example, a higher modified Bhalla score or a higher bronchiectasis score at baseline can be used to identify patients with higher RTE frequency (76, 77). Nonetheless, although CT findings can demonstrate the reversibility of RTE-induced lung abnormalities, CT-detected abnormalities may not differ between RTE and asymptomatic patients (78), that may show a large variability in the resolution of structural abnormalities. This indicates that CT is a reliable tool to monitor new structural changes related to RTE but not to diagnose RTE itself (Figure 4).

Current low and ultra-low CT protocols have further reduced the risk of radiation exposure (7), especially during long-term monitoring of PCF. However, clinicians remain reluctant to use CT for short-term follow-up of RTE treatment, especially in children, who are more sensitive to radiation (79). Further dose reduction could be achieved following the recent introduction of the photon-counting detector CT, a new technology which enables higher resolution at lower dose range (7, 80). Therefore, concerns about radiation exposure may become a minor issue for short-term RTE monitoring.

**Figure 5:**
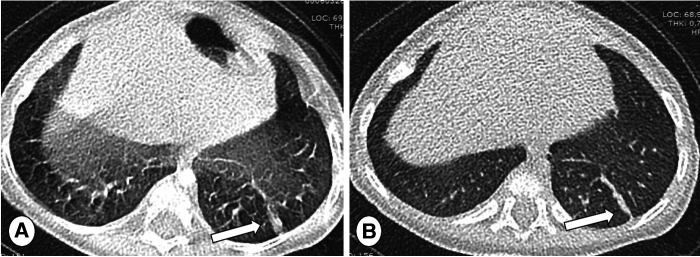
Axial CT of 5 months old patients with CF, thickness 0.7 mm, scanned in single source-mode with pitch 1.2 (**A**) and repeated in double source-mode with pitch 3.2 (**B**). Note that respiratory artifacts are absent using an higher pitch acquisition (**B**), providing a high image quality and facilitating the detection of atelectasis in the left lower lobe (arrows).

Another major advantage of CT is its scan speed: high image-quality – thanks to fast acquisition strategies and iterative reconstructions – can also be achieved in non-collaborative patients, such as infants and young children (81), i.e., adopting high-pitch with dual source acquisitions (Figure 5). This facility reduces the need for sedation or general anesthesia compared with MRI, which requires longer acquisition time. Conversely, CT is more limited than MRI in providing functional information such as that which may relate to perfusion and inflammation. For instance, the use of iodine contrast is needed to obtain perfusion mapping with CT, while non-contrast techniques, described below, are available for MRI.

Finally, CT imaging is far beyond the capability of MRI in terms of automatic quantification of lung abnormality. Automatic scoring systems for CT in PCF have been proposed and tested in large cohorts (82, 83). These tools eliminate the variability related to inter-observer assessment and increase robustness of quantitative data. Other software now focuses on the assessment of airway-artery ratio, the most sensitive outcome parameter of early airways disease in PCF (73, 74).

### Magnetic resonance imaging

Magnetic Resonance Imaging might become the “one-stop-shop” for diagnosing and monitoring RTE in PCF. In facts, thanks to several MRI techniques, information on structure, inflammation and function may be collected during a single examination and without radiation exposure.

**Figure 6:**
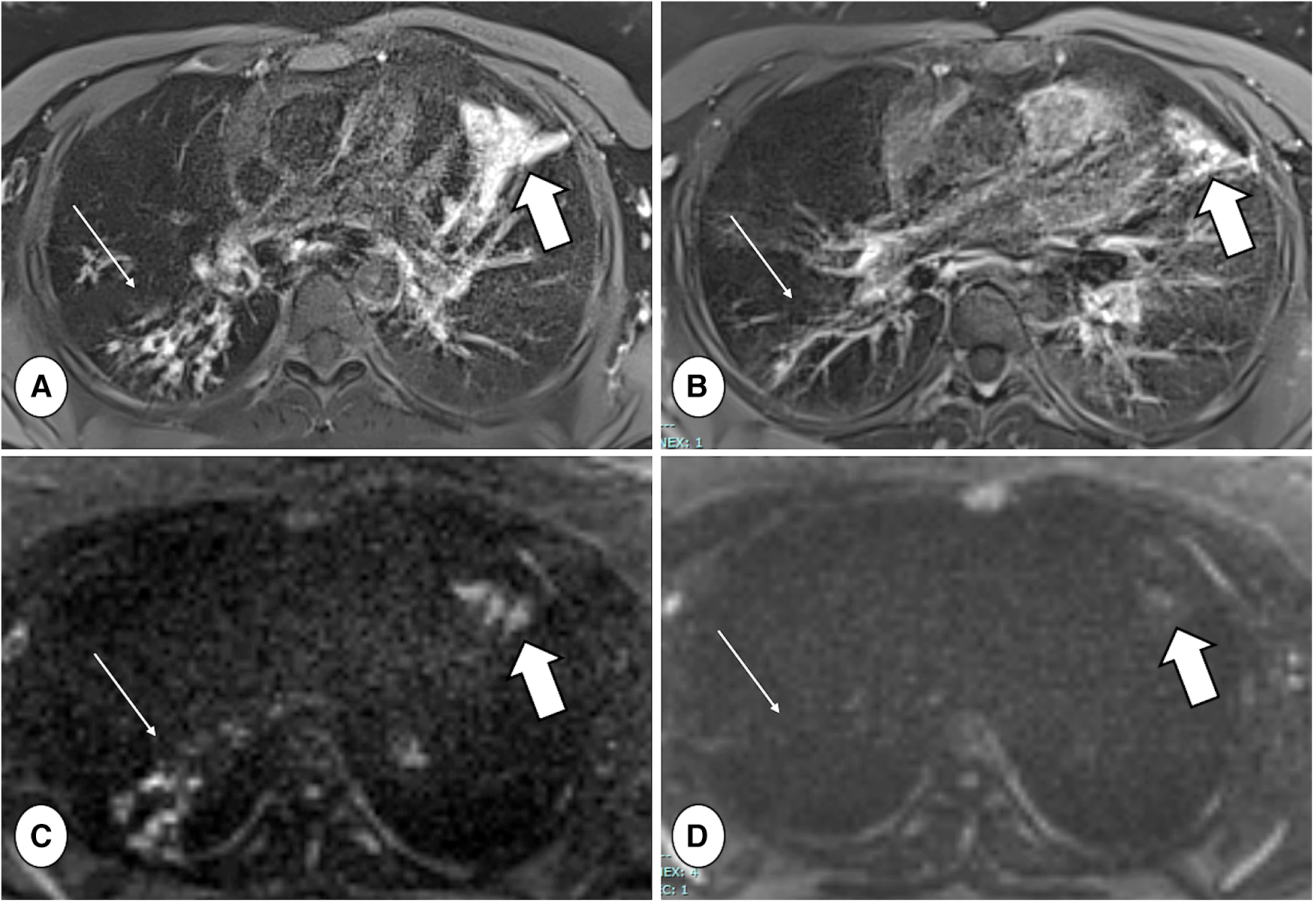
Axial proton density-weighted PROPELLER free-breathing scan (**A,B**) and diffusion-weighted imaging (DWI) (b = 800 mm/s^2^) acquisition (**C,D**), both performed with a 1.5 T MRI system (MAGNETOM avanto, siemens healthineers, enlargen, Germany), in a patient with CF during respiratory tract exacerbation at baseline (**A,C**) and after treatment (**B,D**). Note the reduction in mucus plugs and DWI signal in the right lower lobe between baseline and follow-up (thin arrows), as well as the reduced consolidation and DWI signal in the lingula (thick arrows).

**Figure 7:**
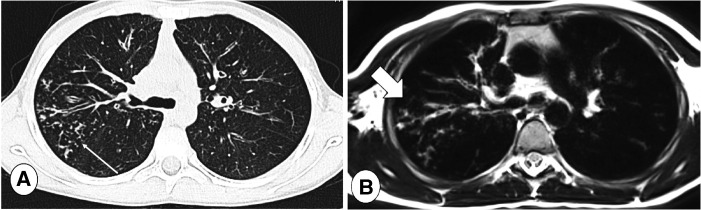
Axial CT (**A**), lung window, and axial T2-weighted MR image (**B**) in a CF patient with tree in bud in the posterior segment of the right upper lobe (thin arrow). Note loss in definition of lung abnormalities in the MR image, where peripheral abnormalities may be not be visible in the MRI sequence (B, thick arrow).

Morphological changes during RTE can be evaluated using conventional sequences such as T1-, T2- and proton density-weighted images (84, 85). According to the patient's age, the protocol can be adapted in terms acquisition type, using end-expiratory triggered sequences in non-cooperative PCF, and breath-hold scans in older patients (Figure 6). Dedicated chest MRI scoring allows a more objective assessment of disease burden, even if the lower resolution of MRI compared with CT remains a limitation. For example, an inferior performance of MRI compared with CT was observed in the morphologic assessment of peripheral alteration, e.g., bronchiectasis and tree in bud, which may be underestimated by MRI sequences (Figure 7) (85).

**Figure 8:**
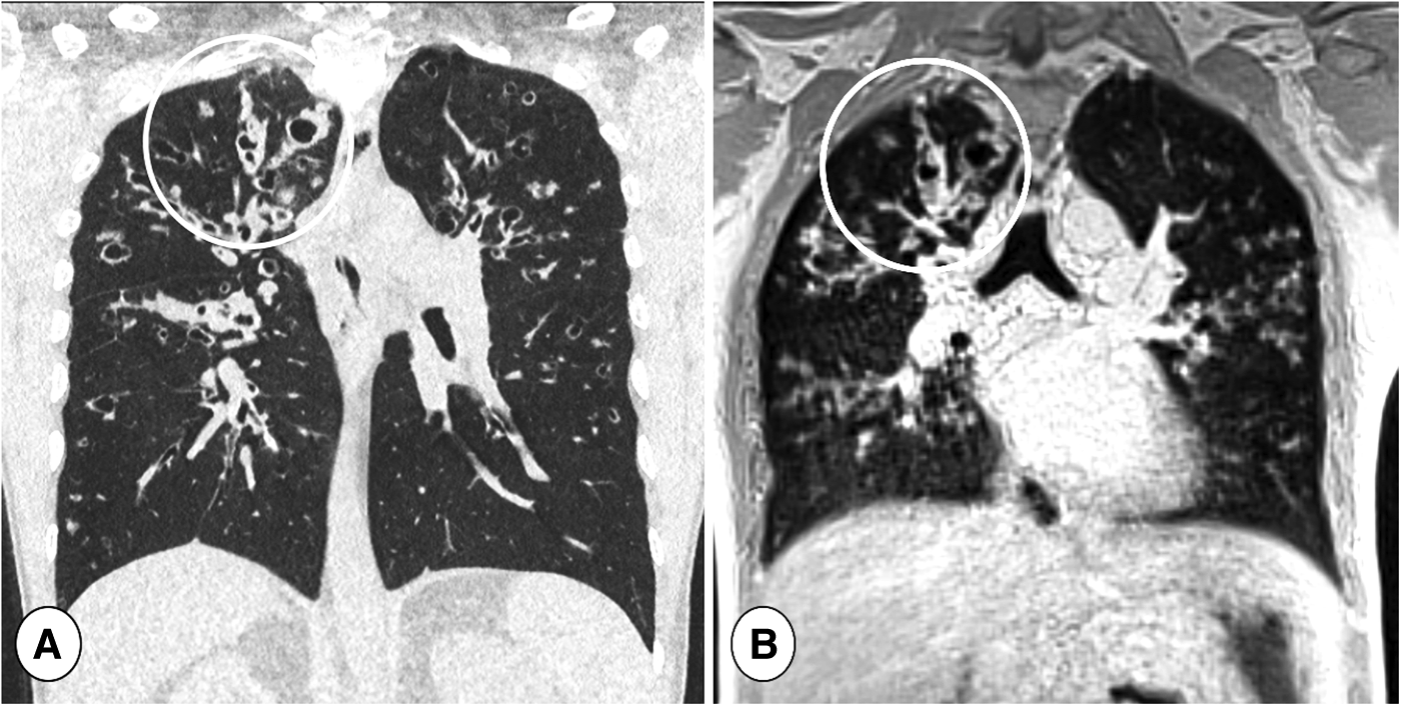
Coronal reformatted CT (**A**) and coronal ultra-short TE (UTE) MR scan (**B**) in a cystic fibrosis patient during respiratory tract exacerbation. Note the high definition of lung abnormalities in both CT and MRI in the right lung (circles).

Nevertheless, recently developed zero echo time or ultrashort echo time (ZTE and UTE) sequences, which are currently the MRI sequences with the best resolution and signal-to-noise ratio (SNR) (Figure 8), were tested with promising results in diffuse lung disease and CF (84, 86, 87). While these sequences allow sub-millimetric isotropic voxel resolution (88), they are limited by a long acquisition time, which are usually around 10 min (86), compared with the few seconds or less of the CT scan. Moreover, in terms of spatial resolution, MRI still does not match CT performance, with best voxel size for MRI of 0.86 mm^3^ against the current 0.2 mm^3^ capability of new Photon Counting CTs (7, 88). Further improvement in image quality is expected from low-field MRI (i.e., 0.55 T) scanners, which should provide higher SNR, as recently shown in diffuse lung diseases (89, 90). However, to date, there are no articles showing the use of low-field MRI scanner for PCF.

**Figure 9:**
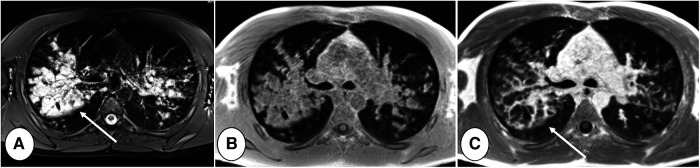
16 years old boy with cystic fibrosis during respiratory tract exacerbation. T2-weighted PROPELLER image (**A**), T1-weighted (**B**) pre and post-contrast (**C**) gradient-echo acquisitions, performed on 1.5 T MRI system (MAGNETOM Avanto, Siemens Healthineers, Enlargen, Germany). Note that in T2-weighted scan bronchial wall cannot be identified in the large bronchiectasis with mucus plugging in the right upper lobe (**A**, arrow). Gadolinium injection allows identification of bronchial wall thickening and to distinguish between bronchial wall and mucus plugs (**C**, arrow).

Inflammation has been studied through diffusion-weighted imaging (DWI) and T2-weighted scans (91–93). Ciet et al*.,* by using a semi-quantitative DWI score on high b-value images, analyzed PCF with and without RTE and those with RTE before and after treatment, interestingly showing significant differences between the groups (91) (Figure 6). Similar results were reported by Benlala et al*.* when quantitatively assessing T2 signal abnormalities during RTE (93). These studies demonstrate the ability of chest MRI to track RTE changes and monitor treatment response. Contrast-enhanced MRI can also be used to evaluate the effects of therapy during RTE. Wielputz et al*.* reported that contrast-enhanced MRI is the most sensitive imaging biomarker of bronchial inflammation during RTE in preschool children with CF (94). Furthermore, using contrast-enhanced MRI, bronchial wall inflammation can be better seen and discriminated from mucus plugs (Figure 9) (94).

Although data on inflammatory activity can be also obtained by positron emission tomography (PET)–CT, the radiation exposure is even higher than with standard CT. As an alternative, PET–MRI may offer the same information at a much lower radiation dose, but there is a lack of RTE data using this technique (1).

Functional information on ventilation are obtained using 3D spoiled-gradient echo, which may show areas of air trapping at end-expiration. In common with CT, this technique can be only used in cooperative patients as it necessitates the ability to breath hold for 5–10 s at both end-inspiration and, especially, end-expiration (95).

**Figure 10:**
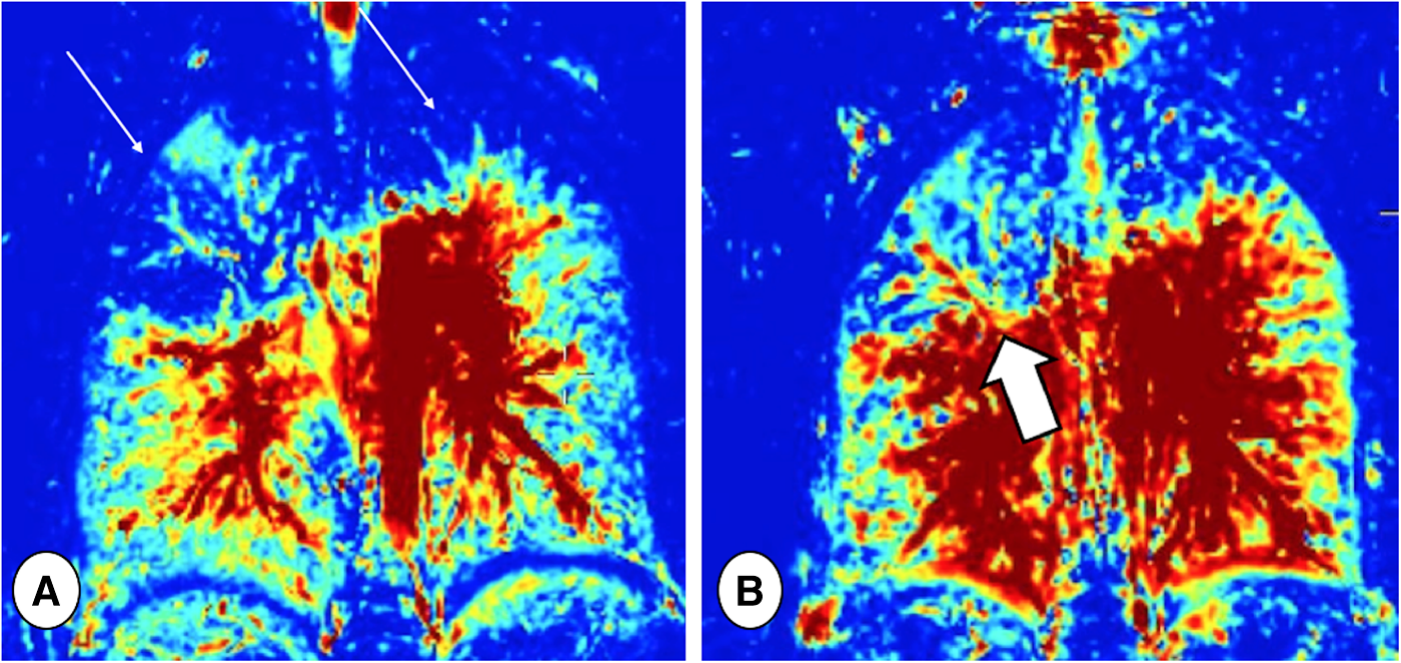
Ventilation maps taken before (**A**) and after (**B**) respiratory tract exacerbation treatment in a patient with CF, obtained by Fourier decomposition MRI. Note the ventilation defects in the upper lobes at baseline (thin arrows), which improved after treatment, especially in the right lung (thick arrow).

Moreover, Fourier decomposition (FD) MRI and derivations such as Phase-Resolved FUnctional Lung (PREFUL) MRI sequences, both ventilation and perfusion can be assessed without intravenous or gaseous inhaled contrast administration (96). PREFUL MRI is also feasible in uncooperative patients, since it is performed in free-breathing conditions (Figure 10). This technique avoids the disadvantages of hyperpolarized-gas MRI in terms of the need for dedicated hardware and the gases costs and storage. Moreover, PREFUL-MRI does not use gadolinium, the use of which is debated because of concerns regarding potential tissue deposition (97). Munisada et al. demonstrated that, during RTE, the ventilation distribution obtained with FD is correlated with hyperpolarized gas imaging and both can monitor functional improvement after therapy (96).

Thus, the ventilation–inflammation–perfusion–structure (VIPS) MRI protocol proposed by Tiddens et al*.* (98) might be a game changer in the diagnosis and management of RTE. However, l studies on chest MRI during RTE are needed though to define a standard MRI protocol for RTE.

In fact, the main factor limiting the use of chest MRI in clinical practice and for RTE monitoring is the need for sedation or general anesthesia in uncooperative patients, both of which require specialized personnel, while the general anesthesia conveys some risks for brain development (99, 100). Higher costs and lower availability of MRI compared with CT, the lack of standardized MRI protocols, and large variability in image quality among MRI vendors may represent further limitations. Finally, only few studies have provided a fully automatic assessment of parenchymal abnormalities using MRI (93), therefore full automation of validated MRI outcomes are needed to facilitate implementation in clinical practice.

### Lung ultrasound

Lung ultrasound is a low-cost, bedside and safe imaging technique used both in pediatric and adult patients with pulmonary diseases. In recent years, interest in this technique has increased, making LUS a valuable point-of-care option.

Lung ultrasound is the method of choice for the diagnosis of pleural diseases, such as pleural effusion and pneumothorax. The technique offers relatively easy access to the costal and diaphragmatic pleura, being also capable of detecting small volumes of pleural effusion. Moreover, despite its use is limited to the lung surface, interest in using LUS to assess diffuse lung disease is increasing, for example, in adult respiratory distress syndrome in intensive care units, Covid-19 pneumonia and interstitial lung diseases (101–105). Unfortunately, limited data are available about the use of LUS to monitor lung status in PCF or evaluate RTE. Nonetheless, preliminary results have shown a good relationship between LUS and CT in assessing structural changes in CF (106, 107). Furthermore, Hassanzad et al*.* demonstrated that LUS is comparable with CT in detecting some pulmonary findings during RTE, especially consolidations and air bronchogram (8). Hence, LUS may be adopted as a complementary imaging tool, as already proposed for CF follow-up, allowing repeatable and easily available examination without any risk to the patient during RTE. However, further multicenter validation studies are needed to evaluate its use in the assessment of CF lung disease.

## Imaging algorithm and recommendations for respiratory tract exacerbations

**Figure 11:**
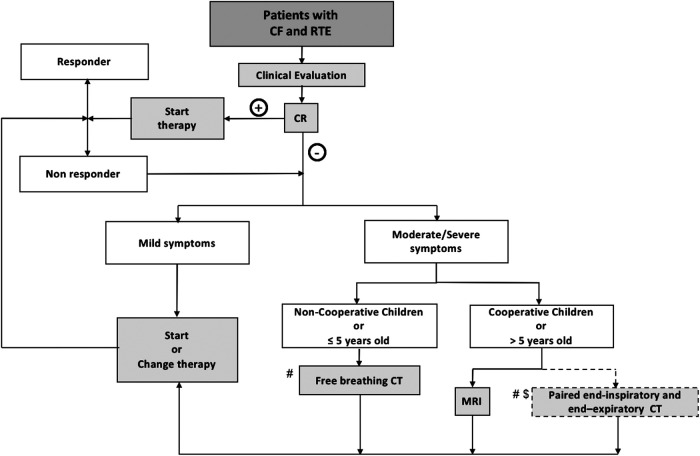
Workflow of the proposed imaging algorithm. CF, Cystic fibrosis; CR, Chest Radiography; CT, computed tomography; MRI, magnetic resonance imaging; RTE: respiratory tract exacerbation; # recent CT or MRI not available (<2 years); $, center with no MRI or no MRI experience; +, positive CR findings; −, negative CR findings.

Based on this literature review and the experience of authors from CF centers, an imaging algorithm for assessing RTE in PCF is proposed (Figure 11).

Given its large availability and low dose-exposure, once the clinical diagnosis is made CR can be performed to look for gross lung abnormalities that may explain the patient's symptoms during RTE and exclude chest complications such as pneumothorax. Comparison with previous CR images is always recommended since it aids the detection of new radiographic abnormalities. In RTE with moderate or severe symptoms (e.g., increased sputum and cough, hemoptysis, dyspnea, chest pain or fever), if the CR outcome is negative or showing no improvement under therapy, further examination is warranted, especially if a recent (<2 years) CT or MRI scan is not available. The choice of the imaging technique depends upon the patient age and compliance. In uncooperative patients or children under 5 years old, a free-breathing CT may be suitable since it is usually conducted without sedation. Fast-acquisition strategies, such as high-pitch with dual source scanning, or restraining tools like vacuum mattresses, often provide sharp image quality without needing sedation or anesthesia (Figure 5). Since free-breathing acquisitions do not allow end-inspiratory and end-expiratory scans to assess air trapping, indirect signs of airway obstruction can be presumed when mosaic attenuation is observed. CT is mainly indicated to exclude major complications, such as empyema or necrotic pneumonia, and is frequently performed with iodine contrast. Computed Tomography is rarely repeated to monitor RTE treatment effects because of concerns about radiation and is thus replaced by CR. In specialized centers with long experience of chest MRI, the latter can also be used in young children under sedation or anesthesia.

In cooperative PCF, generally more than 5 years old, MRI can offer a valid alternative to assess both structural and functional information related to RTE. Chest MRI provides relevant information about ventilation and perfusion, both of which are important in the assessment of RTE. Moreover, MRI can be used repeatedly to monitor the effects of treatment without any limitation in terms of radiation exposure. In centers with no experience of chest MRI, a paired non-contrast-enhanced end-expiratory and contrast-enhanced end-inspiratory CT can be considered.

## Conclusions

Physical examinations and PFTs have several limitations in respect to RTE diagnosis and monitoring. Current sputum and blood biomarkers of infection and inflammation during RTE are not sensitive nor robust enough for the reliable diagnosis of RTE. Therefore, lung imaging is still crucial for diagnosing RTE and monitoring treatment response. While chest radiography can exclude gross abnormalities or complications, it cannot sensitively diagnose new structural changes induced by RTE, especially in PCF with severe lung disease. Computed tomography allows a detailed assessment of RTE-related structural changes and represent the current standard for CF lung disease evaluation. However, short-term CT serial imaging during RTE is limited by the risk of radiation exposure, and it might be only advised with the introduction of ultralow-dose CT protocols. On the other hand, magnetic resonance imaging (MRI) remains behind CT in terms of morphologic assessment capability but, thanks also to recently developed morphological sequences (i.e., zero echo time or ultrashort echo time sequences), could become a one-stop-shop for obtaining both structural and functional information in a single examination. Current limitations of MRI include the need for sedation or anesthesia in uncooperative PCF, the lack of image-quality standardization and the necessity of developing dedicated post-processing tools.
